# Inhibition of *Klebsiella pneumoniae* Growth and Capsular Polysaccharide Biosynthesis by *Fructus mume*

**DOI:** 10.1155/2013/621701

**Published:** 2013-08-26

**Authors:** Tien-Huang Lin, Su-Hua Huang, Chien-Chen Wu, Hsin-Ho Liu, Tzyy-Rong Jinn, Yeh Chen, Ching-Ting Lin

**Affiliations:** ^1^School of Chinese Medicine, China Medical University, No. 91 Hsueh-Shih Road, Taichung 40402, Taiwan; ^2^Division of Urology, Department of Surgery, Taichung Branch, Buddhist Tzu Chi General Hospital, Taichung 40402, Taiwan; ^3^Department of Biotechnology, Asia University, Taichung 41354, Taiwan; ^4^Institute of Biochemistry and Molecular Biology, National Yang-Ming University, Taipei 11221, Taiwan; ^5^Research Institute of Biotechnology, Hungkuang University, Taichung 43302, Taiwan

## Abstract

*Klebsiella pneumoniae* is the predominant pathogen isolated from liver abscess of diabetic patients in Asian countries. With the spread of multiple-drug-resistant *K. pneumoniae*, there is an increasing need for the development of alternative bactericides and approaches to block the production of bacterial virulence factors. Capsular polysaccharide (CPS), especially from the K1 and K2 serotypes, is considered the major determinant for *K. pneumoniae* virulence. We found that extracts of the traditional Chinese medicine *Fructus mume* inhibited the growth of *K. pneumoniae* strains of both serotypes. Furthermore, *Fructus mume* decreased the mucoviscosity, and the CPS produced in a dose-dependent manner, thus reducing bacterial resistance to serum killing. Quantitative reverse transcription polymerase chain reaction analyses showed that *Fructus mume* downregulated the mRNA levels of *cps* biosynthesis genes in both serotypes, possibly by increasing the intracellular iron concentration in *K. pneumoniae*. Moreover, citric acid, a major organic acid in *Fructus mume* extracts, was found to have an inhibitory effect on growth and CPS biosynthesis in *K. pneumoniae*. Taken together, our results indicate that *Fructus mume* not only possesses antibacterial activity against highly virulent *K. pneumoniae* strains but also inhibits bacterial CPS biosynthesis, thereby facilitating pathogen clearance by the host immune system.

## 1. Introduction

*Klebsiella pneumoniae* is an enteric gram-negative bacterium that causes community-acquired diseases, including pneumonia, bacteremia, septicemia, and urinary and respiratory tract infections, particularly in immunocompromised patients [1–4]. In Asian countries, especially in Taiwan and Korea, *K. pneumoniae* is the predominant pathogen responsible for pyogenic liver abscess in diabetic patients [2, 3, 5, 6]. In recent years, reports of *Klebsiella* liver abscess (KLA) in western countries have also been accumulating [6, 7]. Among the virulence factors identified in *K. pneumoniae*, capsular polysaccharide (CPS) is considered as the major determinant for *K. pneumoniae *virulence. Pyogenic liver abscess isolates often carry heavy CPS loads that could protect the bacteria from phagocytosis and killing by serum factors [6–8]. The capsular serotypes of *K. pneumoniae* have been classified into more than 77 known types [9, 10]. In Taiwan, a high prevalence of the K1 and K2 serotypes of *K. pneumoniae* has been documented in liver abscess in diabetes mellitus patients [11]. Extended spectrum *β*-lactamase- (ESBL-) producing *K. pneumoniae*, for which clinical treatment is difficult, has a wide distribution [4, 12]. As a result, there is an urgent need to develop a novel antimicrobial strategy to block the production of virulence factors. 

In accordance with the significance of CPS in the physiology and pathogenesis of *K. pneumoniae*, the biosynthesis of CPS is controlled by a complex network of multiple regulators such as the Rcs system, RmpA, RmpA2, KvhR, KvgAS, and KvhAS [13–16]. Recently, we found that CPS production by *K. pneumoniae* was controlled by external iron and glucose concentrations via the regulation of ferric uptake regulator (Fur) and cAMP-dependent carbon catabolite repression (CCR), respectively [17, 18]. Iron availability has been demonstrated to affect multiple cellular functions such as oxidative stress, energy metabolism, acid tolerance, and virulence factor production in many bacteria [19–22]. Likewise, cAMP signaling has been demonstrated to regulate the expression of various genes encoding carbon metabolism enzymes and virulence factors, such as flagella, fimbriae, protease, exotoxin, and secretion systems [23–32]. These studies indicate that, in response to specific environmental signals, pathogenic bacteria express genes encoding virulence factors that help them to establish a successful infection.

Traditional Chinese medicine (TCM) has been a rich natural source of antimicrobial agents for treating various infectious diseases for more than 4,000 years. Mei, *Prunus mume*, has long played an important role in human diet and health. *Fructus mume*, the smoked fruit of *Prunus mume*, is a TCM that has been used to relieve cough, treat ulceration, and improve digestive function. *Prunus mume* extract has also been shown to inhibit *Helicobacter pylori* infection, which is associated with gastritis and gastric ulcers [33]. In addition, *Fructus mume* extract is a potential candidate for developing an oral antimicrobial agent to control or prevent dental diseases associated with several oral pathogenic bacteria [34–36]. A study involving high-pressure liquid chromatography (HPLC) analysis has also demonstrated that citric acid is the main organic acid in *Fructus mume* extract [35, 36].

In this study, we aimed to assess the antibacterial activity of *Fructus mume* against *K. pneumoniae*, and 2 highly virulent clinical strains, NTUH-K2044 and CG43S3, respectively, belonging to the K1 and K2 serotypes, were used in the following analyses. We found that *Fructus mume* not only possess antibacterial activity against the 2* K. pneumoniae* strains but also reduced bacterial CPS production, thus decreasing the survival rate of bacteria in normal human serum. The regulatory effect of *Fructus mume* on *cps* gene expression in *K. pneumoniae* has also been clarified.

## 2. Materials and Methods

### 2.1. *K. pneumoniae* Strains and Primers

*K. pneumoniae* strains and primers used in this study are listed in Table 1. Bacteria were routinely cultured at 37°C in Luria-Bertani (LB) broth or agar plate.

### 2.2. Preparation of *Fructus mume* Extract

The concentrated herbal medicine,* Fructus mume*, was purchased from Chuang Song Zong Pharmaceutical Co. Ltd. (Kaohsiung, Taiwan) under the good manufacturing practice (GMP) criteria. *Fructus mume* powder was dissolved in LB broth by end-over-end mixing at room temperature for 2 h. The extract of *Fructus mume* was collected by centrifugation (13,000 rpm for 10 min) to remove starch and then filtered by 0.45 *μ*m filter. 

### 2.3. Antibacterial Activity of *Fructus mume *Extract

LB broth or LB broth supplemented with 5, 10, or 20 mg/mL *Fructus mume *extract was initially inoculated with *K. pneumoniae *NTUH-K2044 or CG43S3 (approximate 10^8^ CFU/mL). Cultures were incubated at 37°C, and samples were taken at 0, 2, 6, and 24 h to determine the viable counts (CFU/mL) for each strain. The assay was performed in triplicate, each with triplicate samples.

### 2.4. Assessment of *K. pneumoniae *Mucoviscosity and CPS Production

The mucoviscosity of *K. pneumoniae *was measured by a low speed centrifugation as previously described [37]. Briefly, equal numbers of overnight-cultured bacteria were centrifuged at 6000 g for 5 min. Then, formation of the bacterial pellet could be observed. To further evaluate the CPS amount of *K. pneumoniae*, the bacterial CPS was extracted and then quantified as previously described [38]. The glucuronic acid content, representing the amount of *K. pneumoniae* K1 and K2 CPS, was determined from a standard curve of glucuronic acid (Sigma-Aldrich) and expressed as micrograms per 10^9^ CFU [39].

### 2.5. Quantitative Reverse Transcription Polymerase Chain Reaction (qRT-PCR)

Total RNAs were isolated from early-exponential-phase grown bacteria cells by use of the RNeasy midi-column (QIAGEN) according to the manufacturer's instructions. RNA was DNase-treated with RNase-free DNase I (MoBioPlus) to eliminate DNA contamination. RNA of 100 ng was reverse-transcribed with the Transcriptor First Strand cDNA Synthesis Kit (Roche) using random primers. qRT-PCR was performed in a Roche LightCycler 1.5 Instrument using LightCycler TaqMan Master (Roche). Primers and probes were designed for selected target sequences using Universal ProbeLibrary Assay Design Center (Roche-applied science) and listed in Table 1. Data were analyzed using the real time PCR software of Roche LightCycler 1.5 Instrument. Relative gene expressions were quantified using the comparative threshold cycle 2^−ΔΔCT^ method with 23S rRNA as the endogenous reference.

### 2.6. Bacterial Survival in Serum

Normal human serum, pooled from healthy volunteers, was divided into equal volumes and stored at −70°C before use. Bacterial survival in serum was determined with minor modifications [37]. In brief, LB broth or LB broth supplemented with 5, 10, or 20 mg/mL *Fructus mume *extract was initially inoculated with *K. pneumoniae *NTUH-K2044 or CG43S3 (approximate 10^8^ CFU/mL). After overnight incubation at 37°C, the bacteria were collected, washed twice with phosphate-buffered saline (PBS), and then adjusted to approximately 1 × 10^6^ CFU/mL. The reaction mixture containing 250 *μ*L of the cell suspension and 750 *μ*L of pooled human serum was incubated at 37°C for 15 min. The number of viable bacteria was then determined by plate counting. The survival rate was expressed as the number of viable bacteria treated with human serum compared to the number of pretreatment. The assay was performed in triplicate, each with triplicate samples. 

### 2.7. Streptonigrin Sensitivity

To measure bacterial susceptibility to the iron-activated antibiotic streptonigrin, overnight grown *K. pneumoniae *were 1 : 10 diluted in LB or LB broth supplemented with different concentrations of *Fructus mume* extract. After 2 h incubation with or without streptonigrin (2 *μ*g/mL) at 37°C with agitation, aliquots (5 *μ*L) of cultures serially diluted tenfold in LB broth were spotted onto LB agar. The plates were incubated at 37°C overnight and photographed.

### 2.8. Statistical Method

An unpaired *t*-test was used to determine the statistical significance, and values of *P* < 0.05 and *P* < 0.01 were considered significant. Each sample was assayed in triplicate, and the mean activity and standard deviation are presented.

### 2.9. Ethics Statement

For isolation of normal human serum from healthy volunteers, the procedure and the respective consent documents were approved by the Ethics Committee of the China Medical University Hospital, Taichung, Taiwan. All healthy volunteers provided written informed consent.

## 3. Results

### 3.1. *Fructus mume* Inhibits the Growth of *K. pneumoniae*

**Figure 1:**
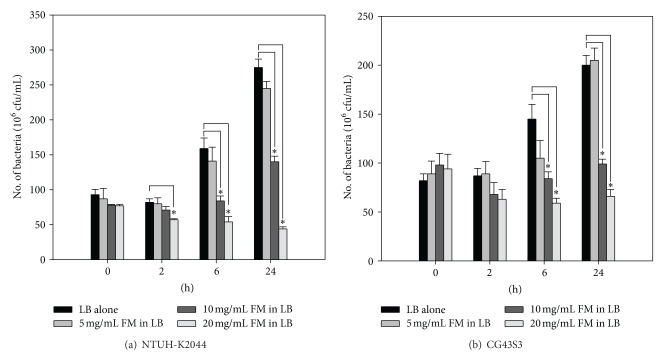
Antibacterial activity of the *Fructus mume* extract against *K. pneumoniae*. The addition of different concentrations of *Fructus mume* extract (FM), as indicated, to LB broth affects the growth of *K. pneumoniae* NTUH-K2044 (a) or CG43S3 (b). LB broth only inoculated with the bacteria serves as a negative control. *, *P* < 0.05 compared to the indicated group.

To examine the antibacterial activity of *Fructus mume*, *K. pneumoniae* NTUH-K2044 and CG43S3 were cocultured with increasing amounts of *Fructus mume* extract, and bacterial growth was monitored by plate counting. As shown in Figure 1, compared to the results for the control groups, addition of 5 mg/mL *Fructus mume* extract to LB broth did not obviously influence the growth of the 2 strains at any time interval, while the addition of 10 or 20 mg/mL *Fructus mume* extract caused significant growth reduction after 6 and 24 h incubations. This inhibitory effect of *Fructus mume* extract is dose dependent. Besides, *Fructus mume* extract appeared to exert a stronger growth inhibitory effect in the case of NTUH-K2044 than in the case of CG43S3. Bactericidal activity of the 20 mg/mL *Fructus mume* extract was observed for NTUH-K2044 but not CG43S3. Additionally, the pH values of LB broth supplemented with 5, 10, and 20 mg/mL FM extract were ∼5.0, 4.4, and 3.6, respectively.

### 3.2. *Fructus mume* Reduces the Biosynthesis of CPS

**Figure 2:**
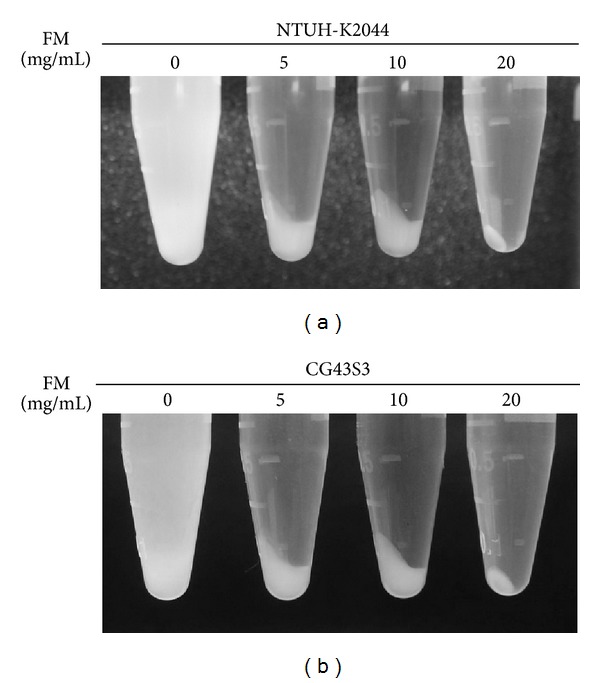
*Fructus mume *reduces* K. pneumoniae *mucoviscosity. Different concentrations of *Fructus mume* extract (FM), as indicated, were added to LB broth inoculated with *K. pneumoniae* NTUH-K2044 (a) or CG43S3 (b). After overnight incubation at 37°C, the bacterial mucoviscosity was assessed by a low speed centrifugation. LB broth only inoculated with the bacteria serves as a negative control.

In the case of *K. pneumoniae* strains, high mucoviscosity, resulting from a large amount of surface CPS, has been correlated with increased pathogenicity [8, 40]. To investigate whether *Fructus mume* affects this mucoviscosity, NTUH-K2044 and CG43S3 were, respectively, cocultured with increasing amounts of *Fructus mume* extract. After 24 h of incubation, the sedimentation test revealed that the addition of *Fructus mume* extract to LB broth obviously decreased mucoviscosity in the case of 2 *K. pneumoniae* strains; *K. pneumoniae* cocultured with *Fructus mume* extract formed a compact pellet after centrifugation, while the control group could not be pelleted down (Figure 2). Since the addition of 5 mg/mL *Fructus mume* extract did not influence bacterial growth (Figure 1), we suggest that *Fructus mume* affected the mucoviscosity by regulating the biosynthesis of CPS. As shown in Table 2, in comparison with the control group, NTUH-K2044 produced a larger amount of CPS, approximately 1.55 fold, than that of CG43S3. The addition of *Fructus mume* extract reduced CPS production in both strains in a dose-dependent manner. Moreover, *Fructus mume* extract appeared to exert a stronger inhibitory effect on NTUH-K2044 than on CG43S3 (Table 2).

### 3.3. *Fructus mume* Reduces the Serum Resistance Activity of* K. pneumoniae*

**Figure 3:**
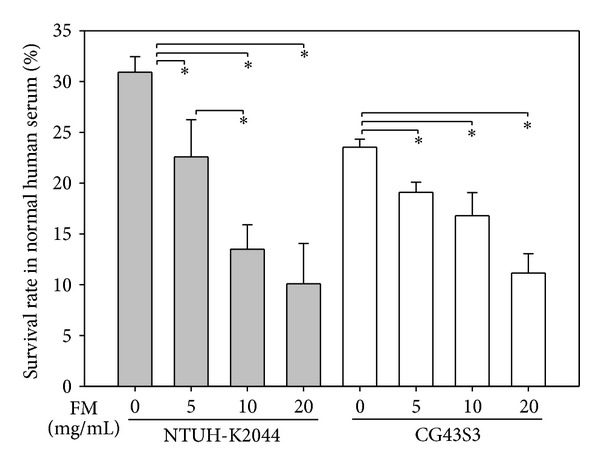
Effect of *Fructus mume* on *K. pneumoniae* susceptibility to normal human serum. Different concentrations of *Fructus mume* extract (FM) were added to LB broth inoculated with *K. pneumoniae* NTUH-K2044 (a) or CG43S3 (b), as indicated in the margin. After overnight incubation at 37°C, the bacterial serum resistance was determined. LB broth only inoculated with the bacteria serves as a negative control. *, *P* < 0.05 compared to the indicated group.

CPS plays a crucial role in the resistance of *K. pneumoniae* to serum killing. Since the *Fructus mume* extract decreased the CPS production of *K. pneumoniae*, the effect of the extract on bacterial serum resistance was further analyzed. NTUH-K2044 or CG43S3 was, respectively, cocultured with 5, 10, or 20 mg/mL *Fructus mume* extract, and the bacteria were then collected, washed, and subjected to incubation with pooled human sera. After 15 min of incubation, the survival rate of the bacteria was determined by plate counting. As shown in Figure 3, *Fructus mume* extract obviously decreased the serum resistance activity of NTUH-K2044 and CG43S3 in a dose-dependent manner, possibly due to reduced CPS production by the bacteria.

### 3.4. Effect of* Fructus mume* on *cps* Transcription

**Figure 4:**
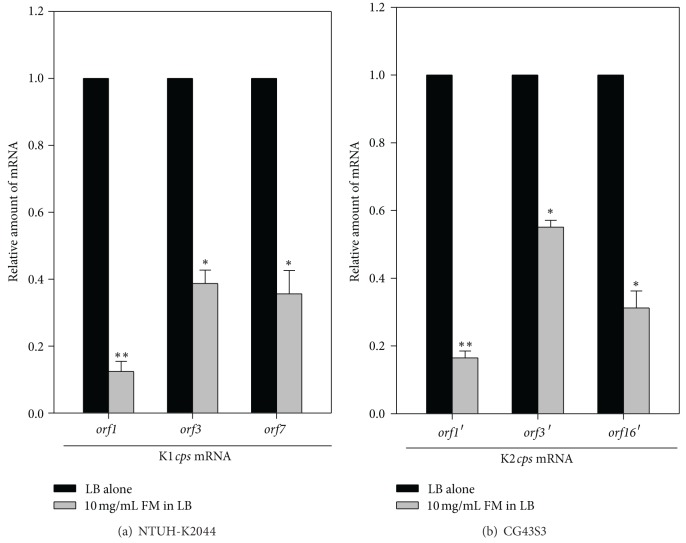
*Fructus mume *downregulates *cps* transcription. qRT-PCR analyses of the expression of the K1 *cps* genes (*orf1*, *orf3*, and *orf7*) in NTUH-K2044 (a) or K2 *cps* genes (*orf1*′, *orf3*′, and *orf16*′) in CG43S3 (b) in LB or LB broth supplemented with 10 mg/mL *Fructus mume* extract (FM). *, *P* < 0.05 and **, *P* < 0.01 compared to LB alone.

The biosynthesis of *K. pneumoniae* K1 and K2 CPS is controlled by 20 and 17 genes, respectively [41, 42]. Both the K1 and K2* cps *gene clusters contain 3 transcriptional units: *orf1-2*, *orf3-6*, and *orf7-20* in the K1 *cps *gene cluster and *orf1*′*-2*′, *orf3*′*-15*′, and *orf16*′*-17*′ in the K2 *cps* gene cluster [41, 42]. To investigate how *Fructus mume* extract affects the biosynthesis of *K. pneumoniae* CPS, NTUH-K2044 and CG43S3 were cocultured with 10 mg/mL *Fructus mume* extract, and the mRNA levels of the 3 transcripts belonging to the K1 or K2 *cps* gene cluster were measured by qRT-PCR. As shown in Figure 4, compared to the control group, the addition of 10 mg/mL *Fructus mume* extract obviously reduced the mRNA levels of all the *cps* transcripts, suggesting that *Fructus mume* regulated CPS biosynthesis at the transcriptional level.

### 3.5. *Fructus mume *Reduces CPS Biosynthesis by Regulating* K. pneumoniae *Intracellular Iron Concentration

**Figure 5:**
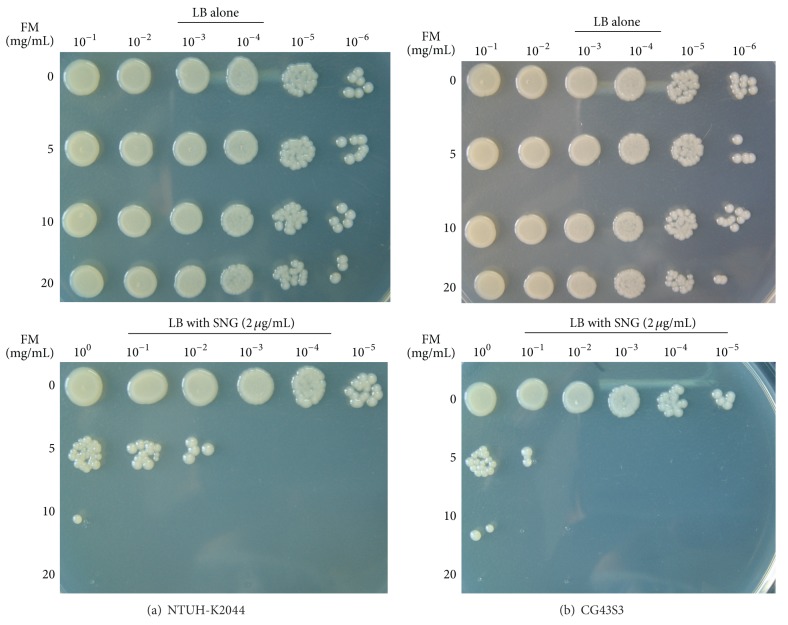
*Fructus mume* increases *K. pneumoniae* susceptibility to streptonigrin.* K. pneumoniae* NTUH-K2044 (a) or CG43S3 (b) cocultured with different concentrations of *Fructus mume* extract (FM) were grown in LB alone or LB that supplemented with 2 *μ*g/mL streptonigrin (SNG) incubated for 2 h. Then, tenfold serial dilutions were spotted onto an LB agar to observe the colony formation.

We have previously demonstrated that iron depletion activated CPS production in *K. pneumoniae* at the transcriptional level [18]. To analyze whether iron is involved in *Fructus mume*-regulated CPS production, we assessed intracellular iron levels in *K. pneumoniae* cocultured with increasing amounts of *Fructus mume* extract, using the iron-activated antibiotic streptonigrin, which requires iron for its bactericidal action that causes DNA degradation [43]. As shown in Figure 5, when NTUH-K2044 or CG43S3 was grown in LB broth only, the bacteria exhibited a streptonigrin-resistant phenotype. However, the streptonigrin susceptibility of the bacteria increased upon coculturing with increasing amounts of *Fructus mume* extract, suggesting that *Fructus mume* increased the intracellular level of free iron in a dose-dependent manner in *K. pneumoniae*. 

### 3.6. Citric Acid Inhibits *K. pneumoniae* Growth and CPS Biosynthesis

**Figure 6:**
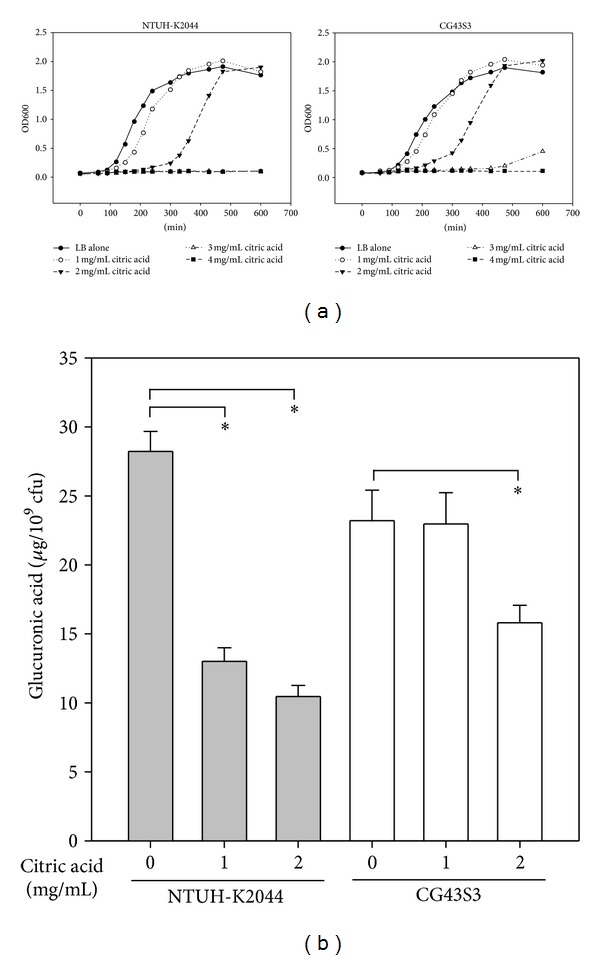
Citric acid inhibits *K. pneumoniae* growth and CPS levels. *K. pneumoniae* NTUH-K204 or CG43S3 cocultured with different concentrations of citric acid, as indicated, and the growth curve (a) as well as the CPS levels (b) were measured. Bacteria were grown in LB broth at 37°C with agitation. *, *P* < 0.05 compared to the indicated group.

Citric acid has been demonstrated to be the main organic acid in *Fructus mume* extract [35]. To examine if citric acid plays a role in the inhibitory effects of *Fructus mume* extract on *K. pneumoniae *growth and CPS production, NTUH-K2044 and CG43S3 were cocultured with various concentrations of citric acid, and then the growth curves of the bacteria were monitored. The result showed that citric acid obviously reduced the growth of NTUH-K2044 and CG43S3 in a dose-dependent manner (Figure 6(a)). Furthermore, CPS production in the 2 *K. pneumoniae* strains also decreased when citric acid was added to the growth medium (Figure 6(b)). These results indicated that citric acid is an active component of *Fructus mume* extract that has bactericidal activity and downregulates CPS biosynthesis in* K. pneumoniae*.

## 4. Discussion

Since the 1980s, *K. pneumoniae* is emerging as an important pathogen in both community and hospital settings [44]. In the hospital environment, due to the extensive use of antibiotics, multiple drug resistance has been increasingly observed in *K. pneumoniae*, especially in ESBL-producing strains. Carbapenems are considered to be the preferred agents for the treatment of serious infections caused by ESBL-producing *K. pneumoniae* because of their high stability with respect to *β*-lactamase hydrolysis and the observed retained susceptibility of ESBL producers [45]. However, *K. pneumoniae* isolates resistant to carbapenems have been reported worldwide since the 2000s [46–49]. The emergence of carbapenem-resistant enterobacteria is worrisome because the option for antimicrobial treatment is further restricted. In this study, we screened a series of TCMs for the identification of new antibacterial agents (data not shown) and then focused on *Fructus mume*, the smoked fruit of *Prunus mume,* which has been demonstrated to efficiently inhibit *H. pylori *and other oral bacteria [33–36]. As shown in Figure 1, *Fructus mume* extract was found to have antibacterial activity against clinically isolated *K. pneumoniae* strains. 

Clinically isolated *K. pneumoniae *strains usually produce a large amount of CPS, which confers not only a mucoid phenotype to the bacteria but also resistance to engulfment by phagocytes and to serum bactericidal factors [40, 50]. The degree of mucoviscosity has also been positively correlated with the successful establishment of infection [51, 52]. Among the 77 described capsular types of *K. pneumoniae*, K1 and K2 serotypes are highly virulent in experimental infection in mice and are often associated with severe infections in humans and animals [52–55], particularly with the tight association between the 2 serotypes and liver abscess [11, 56]. As shown in Figure 2 and Table 2, in *K. pneumoniae* strains of both the K1 and K2 serotypes, *Fructus mume* extract significantly reduced bacterial hypermucoviscosity and CPS levels and thus may result in the decreased resistance of *K. pneumoniae *to serum killing (Figure 3). On the other hand, we also found that heat inactivated serum did not have obvious bactericidal effects on *K. pneumoniae* (data not shown), suggesting an importance of the complement system.

Both prokaryotic and eukaryotic cells in natural environments are constantly challenged by various environmental stresses. *K. pneumoniae*, like many gastrointestinal pathogens, needs to penetrate the gastric acid barrier, face the challenge of the immune system, and cope with the limited supply of oxygen and nutrition to effect colonization and infection [57, 58]. Therefore, bacteria activate or repress the expression of virulence genes to adapt to environmental stimuli [59–62]. In *K. pneumoniae*, CPS biosynthesis is regulated by multiple environmental stimuli and protein regulators. Our previous studies have shown that ferric ions can repress *K. pneumoniae *CPS production through Fur regulation [18, 63]. Under iron-repletion conditions, Fur-Fe(II) can tightly repress *cps* transcription, resulting in lowered CPS production in *K. pneumoniae* [18]. To further investigate how *Fructus mume* reduces CPS levels in *K. pneumoniae*, we performed qRT-PCR analyses and found that CPS reduction was regulated at the transcriptional level (Figure 4). All 3 transcripts of K1 or K2 *cps* genes were obviously downregulated, especially *orf1*/*orf1*′ encoding GalF, a putative UDP-glucose pyrophosphatase (Figure 4). In addition, the streptonigrin sensitivity assay also indicated that *Fructus mume* could increase the intracellular iron levels of *K. pneumoniae *(Figure 5), implying that ferric iron and Fur participate in *Fructus mume*-mediated CPS reduction. Iron is essential to most bacteria for growth and reproduction, but iron overloading would lead to the formation of undesired reactive oxygen species (ROS) by the Fenton reaction to damage DNA or other biological macromolecules [64]. Although how *Fructus mume* affects bacterial iron-uptake remains unknown, the increase in intracellular iron leading to the formation of undesired ROS may account for part of its bactericidal activity. 

The ingredients of *Prunus mume* extract are organic acids, including citric acid (the main ingredient), tartaric acid, oxalic acid, and other unknown components [35, 36]. Although the acidic components of *Prunus mume* showed some antibacterial activity, it is less compared to that of the original extract, implying that other active components possess considerable antibacterial activity [36]. As seen in Figure 6, we also showed that citric acid could inhibit growth and CPS production in* K. pneumoniae*. The acidic property may be one of the antibacterial mechanisms of *Fructus mume*; however, there should be other active components, since LB broth supplemented with 20 mg/mL *Fructus mume*, pH ∼3.6, has stronger bactericidal activity than LB broth adjusted to pH 3.5 using HCl (data not shown). For future clinical application, more studies are required to determine the active components of* Fructus mume* and its mechanism of action.

## 5. Conclusion

To our knowledge, this study is the first to report the antibacterial activity of *Fructus mume* against highly virulent *K. pneumoniae *isolates. Moreover, we found that *Fructus mume* reduced *K. pneumoniae* CPS biosynthesis at the transcriptional level, possibly by regulating the intracellular iron concentration of the bacteria, thereby helping the host immune system eliminate the pathogen.

## Figures and Tables

**Table:** (a)

Strains	Descriptions	Reference or source
*K. pneumoniae *		
NTUH-K2044	K1 serotype	From Dr. Jin-Town Wang
CG43S3	K2 serotype	From Dr. Hwei-Ling Peng

**Table:** (b)

Primer	Sequence (5′→3′)	TaqMan probes	Target
RT03	CGTCATCCAGACCAAAGAGC	83	*orf1 *in K1 *cps* gene cluster
RT04	CCGGTTTTTCAATAAACTCGAC		*orf1*′ in K2 *cps* gene cluster
RT134	TACCGGGACAGAGAATGAGC	78	*orf3 *in K1 *cps* gene cluster
RT135	TAACTGGCCAACCCAAGGT		
RT136	CGTTTTATGGTAATGTTCTCCTCA	26	*orf7 *in K1 *cps* gene cluster
RT137	TCTGCCCATAACCTCGAAAG		
RT05	CGATGACCGGCTTTTTAATG	83	*orf3*′ in K2 *cps* gene cluster
RT06	CTAGCGGAGATTTGGTACTGC		
RT07	CAGTCCACCTTTATTCCGATTG	67	*orf16*′ in K2 *cps* gene cluster
RT08	AGGTACGACCCCGACTGG		

**Table 2:** Quantification of CPS amount of *K. pneumoniae *strains cocultured with *Fructus mume* extract.

*Fructus mume* (mg/mL)	CPS amount^a^ (% relative to the control group)	
	NTUH-K2044	CG43S3
0	27.33 ± 3.45 (100)	17.61 ± 1.53 (100)
5	15.46 ± 2.18 (56.6)	13.15 ± 1.0 (74.7)
10	11.42 ± 1.69 (41.8)	10.86 ± 1.05 (61.7)
20	ND^b^	ND^b^

^a^glucuronic acid content (*μ*g/10^9^ cfu).

^b^ND: not determined.
